# Targeting Nanostrategies for Imaging of Atherosclerosis

**DOI:** 10.1155/2021/6664471

**Published:** 2021-03-31

**Authors:** Angela Costagliola di Polidoro, Agnese Grassia, Francesca De Sarno, Paolo Bevilacqua, Valentina Mollo, Eugenia Romano, Maria Donata Di Taranto, Giuliana Fortunato, Umberto Marcello Bracale, Liberatore Tramontano, Tommaso Claudio Diomaiuti, Enza Torino

**Affiliations:** ^1^Department of Chemical, Materials Engineering & Industrial Production, University of Naples Federico II, Piazzale Tecchio 80, Naples 80125, Italy; ^2^Center for Advanced Biomaterials for Health Care (CABHC), Fondazione Istituto Italiano di Tecnologia (IIT)CRIB, Largo Barsanti e Matteucci 53, Naples 80125, Italy; ^3^IRCCS SDN, via Gianturco 113, Naples, Italy; ^4^Department of Molecular Medicine and Medical Biotechnologies, University of Naples Federico II, via S. Pansini 5, Naples 80131, Italy; ^5^CEINGE Advanced Biotechnologies S.C. A R.L., via Gaetano Salvatore 486, Naples 80145, Italy; ^6^Department of Public Health, University of Naples Federico II, via S. Pansini 5, Naples 80131, Italy; ^7^Interdisciplinary Research Center on Biomaterials (CRIB), Piazzale Tecchio 80, Naples 80125, Italy

## Abstract

Despite the progress in cardiovascular research, atherosclerosis still represents the main cause of death worldwide. Clinically, the diagnosis of Atherosclerotic Cardiovascular Disease (ASCVD) relies on imaging methodologies including X-ray angiography and computed tomography (CT), which however still fails in the identification of patients at high risk of plaque rupture, the main cause of severe clinical events as stroke and heart attack. Magnetic resonance imaging, which is characterized by very high spatial resolution, could provide a better characterization of atherosclerotic plaque (AP) anatomy and composition, aiding in the identification of “vulnerable” plaques. In this context, hydrogel matrices, which have been demonstrated able to boost relaxometric properties of Gd-based contrast agents (CAs) by the effect of Hydrodenticity, represent a valuable tool towards the precision imaging of ASCVD improving the performance of this class of CAs while reducing systemic toxicity. In particular, hydrogel nanoparticles encapsulating Gd-DTPA can further contribute to providing CA-specific accumulation in the AP by nanoparticle surface decoration triggering an active targeting of the AP with the overall effect of allowing an earlier and more accurate diagnosis. In this work, we tested crosslinked Hyaluronic Acid Nanoparticles (cHANPs) in the complex environment of human atherosclerotic plaque. In addition, the surface of cHANPs was decorated with the antibody anti-CD36 (Ab36-cHANPs) for the active targeting of AP-associated macrophages. Results demonstrate that the Hydrodenticity of cHANPs and Ab36-cHANPs is preserved in this complex system and, preliminarily, that interaction of these probes with the AP is present.

## 1. Introduction

To date, Atherosclerotic Cardiovascular Disease (ASCVD) represents one of the leading causes of death daily worldwide. However, it is characterized by an increasing incidence that is difficult to determine accurately since ASCVD is a predominantly asymptomatic condition [1].

Atherosclerosis is an inflammatory pathology that affects the intima and media layers of arteries of medium and large size and is characterized by the formation of typical Atherosclerotic lipid Plaques (AP) that cause the narrowing of the vessel lumen. During atherogenesis, the process of AP formation, specific changes occur in the endothelial lining of the vessel allowing the invasion by inflammatory and immune cells (macrophages and T cells) [2]. The consequent inflammation plays a key role in the pathological process [3] triggering the migration and proliferation of Smooth Muscle Cells (SMCs) from the media that causes a blood flow reduction with different complications [4, 5]. Nevertheless, the degree of stenosis of the vessel is only partially related to the risks associated with this pathology, with the AP composition being the main cause of instability phenomena that lead to the manifestation of clinical symptoms such as stroke, heart attack, and ischemia [6]. Indeed, the stability of APs is influenced by many factors, such as the lipid core, the thickness of the fibrous cap, and the inflammation within the cap. The rupture is most likely associated with “vulnerable plaques” and may cause acute clinical events (e.g., stroke and heart attack) and thrombosis [7–9].

From a clinical point of view, atherosclerosis remains silent for many decades [5, 9]. Diagnosis relies on cardiovascular imaging that has been focused mainly on the determination of the degree of luminal stenosis that, as said, is only an indirect indicator of the pathological process that allows a later diagnosis [10]. Standard imaging techniques, such as X-ray angiography, computed tomography (CT), and Positron Emission Tomography (PET), allow displaying different morphological or functional features of the atherosclerotic lesion. However, they are still failing in the identification of patients at risk of coronary plaque rupture and the evaluation of the efficacy of administered therapies [11, 12].

In this context, Magnetic Resonance Imaging (MRI) represents a promising alternative to other imaging techniques since it is characterized by a very high spatial resolution that might allow to better characterize AP anatomy and thus identify AP composition without the use of ionizing radiations [13]. To improve MRI sensitivity, contrast agents (CAs) can be intravenously injected and different components of the AP may be discriminated. However, CAs suffer from general drawbacks such as the requirement of long scan times, rapid renal clearance, and toxicity due to their nonspecific biodistribution. These drawbacks are particularly relevant for ASCVD applications since the CAs in clinical practice are not able to accumulate selectively in the AP at a concentration guaranteeing the level of detail required for early diagnosis and the anatomical characterization of APs.

Therefore, noninvasive and early identification of high-risk patients and improved accuracy of patient treatment response can be achieved by providing improved performance of CAs on one hand and their sufficient accumulation in the AP on the other.

The combination of hydrogel matrices and Gd-based CAs has been demonstrated to be able to boost relaxometric properties of Gd-chelates, the most common CAs in clinical MRI, through Hydrodenticity [14–16]. Hydrodenticity refers to hydrogel matrices' ability to improve the hydration of molecules of Gd-chelates through the formation of a complex equilibrium between the elastodynamics of polymer chains and water osmotic pressure, providing a boosting of Gd relaxometric properties up to 12 times [14]. In addition, an active targeting approach can be used to exploit active physiological or pathological mechanisms of cellular internalization to promote the selective accumulation of CAs inside the AP [17].

In this scenario, hydrogel-based nanoparticles encapsulating Gd-chelates have great potential since it is possible to combine the numerous advantages of nanoformulated contrast agents with the unique property of Hydrodenticity, conferring the ability to boost Gd-chelates relaxivity without any modification of the chelated ion as approved in the clinical practice, thus aiding the nanoformulation in the process of clinical evaluation and approval. Indeed, it has been reported that the combination of contrast agents with nanoparticles can be brought to the market by the premarket notification, or 510(k), process [18].

In addition, nanoparticles are able to protect Gd-chelates from transmetalation, reducing toxicity, prolong their circulation time, and provide an increased surface by their high surface-to-volume ratio, which can be decorated ad hoc to exploit an active targeting approach [19–21].

Despite the great variety of hydrophilic polymers available, hyaluronic acid (HA) represents a natural candidate for the selective delivery of CAs to the AP since it has been demonstrated to be a substrate for many receptors particularly abundant in the AP as CD44 and ICAM [22]. Moreover, HA has been proven to prolong blood circulation avoiding recognition by the reticuloendothelial system (RES) as a hydrophilic polymer. In 2017, Beldman et al. [23] proposed radiolabelled Hyaluronic Acid Nanoparticles as a selective imaging agent for PET imaging of atherosclerosis, demonstrating an improved uptake of nanoparticles by macrophages in vitro and the accumulation of nanoparticles in the atherosclerotic plaque in vivo. In addition, the discovery that inflammation drives plaque growth and rupture additionally suggests that the immune system might be an interesting diagnostic target [24]. Specifically, the macrophage scavenger receptor CD36 is present on the surface of macrophages and is implicated in the formation of foam cells. Indeed, the process that starts with the uptake of oxidized Low-Density Lipoprotein (oxLDL) by macrophages causes the upregulation of CD36 which is called the “eat me signal.” In addition, oxLDL via CD36 inhibits macrophage migration exerting a macrophage-trapping mechanism in the atherosclerotic lesion. For these reasons, CD36 has gained increasing attention as an immune system target in the latter years [25]. Lipinsky et al. [26] demonstrated that the surface functionalization of Gd-loaded lipid-based nanoparticles with the antibody anti-CD36 significantly improved in vitro the uptake of nanoparticles by macrophages and the MR signal measured ex vivo on human atherosclerotic plaques.

In this work, we explored the ability of anti-CD36 decorated crosslinked Hyaluronic Acid Nanoparticles (Ab36-cHANPs) encapsulating Gd-DTPA to preserve the property of Hydrodenticity in a complex environment such as the human AP removed by surgical endarterectomy. The surface functionalization with the antibody anti-CD36 in combination with the double function of HA as hydrophilic polymer boosting Gd-DTPA relaxivity by Hydrodenticity and intrinsic target of the AP confer to the formulation a great potential for the active targeting and boosted MRI of atherosclerosis.

## 2. Materials and Methods

### 2.1. Materials

Sodium hyaluronate (*M*_*w*_ = 50000 Da) was purchased from Bohus BioTech (Strömstad, Sweden). Diethylenetriaminepentaacetic acid gadolinium(III) dihydrogen salt hydrate Gd-DTPA (*M*_*w*_ = 547.57 Da), acetone (CHROMASOLV, for HPLC, ≥99.8%; molecular formula CH_3_COCH_3_; *M*_*w*_ = 58.08 Da), ethanol (ACS reagent, ≥99.5% (200 proof), absolute; molecular formula CH_3_CH_2_OH; *M*_*w*_ = 46.07 Da), divinyl sulfone (DVS) (contains <650 p.p.m. hydroquinone as inhibitor; purity 97%; density 1.117 g/ml at 25°C (lit.) molecular formula C_4_H_6_O_2_S, *M*_*w*_ = 118.15 Da), streptavidin (*M*_*w*_ = 60000 Da), sodium hydroxide NaOH (ACS reagent, ≥97.0%, *M*_*w*_ = 40.00 Da), (3-dimethylaminopropyl)-N'-ethylcarbodiimide hydrochloride EDC (molecular formula C_8_H_17_N_3_ HCl; *M*_*w*_ = 191.70 Da), *N*-hydroxysuccinimide NHS (molecular formula C_4_H_5_NO_3_; *M*_*w*_ = 115.09 Da), and Fomblin® (perfluoropolyether) were all purchased from Merck KGaA (Germany). QuantiPro™ BCA Assay Kit was purchased from Sigma Aldrich (St. Louis, MI, USA). The rabbit anti-human polyclonal antibody to human CD36 and its biotinylated form is purchased from Invitrogen (Thermo Fisher Scientific, USA). Glutaraldehyde (25% aqueous solution; EM Grade, molecular formula OCHCH_2_CH_2_CH_2_CHO, CAS no. 111-30-8) paraformaldehyde (16% aqueous solution, EM Grade, methanol-free solution, specific gravity: 1.09, molecular formula: HCHO), sodium cacodylate buffer (prepared from sodium cacodylate trihydrate, F.W. 214.02), potassium ferrocyanide (ACS reagent, molecular formula K3Fe(CN)6, F.W. 329.25), osmium tetroxide (4% aqueous solution, molecular formula OsO_4_, F.W. 254.20), uranyl acetate (4% aqueous solution ACS reagent, molecular formula UO_2_(OCOCH_3_)_2_·2H_2_O, F.W. 424.14), Spurr epoxy resin (low-viscosity kit: Nonenyl Succinic Anhydride (NSA), EM grade, DER 736, specific gravity: 1.14, ERL 4221 Cycloaliphatic Epoxide Resin specific gravity (H_2_O = 1), DMAE 2-dimethylaminoethanol C_4_H_11_NO, F.W. 89.14, specific gravity: 0.883–0.888) were purchased from Electron Microscopy Sciences. The Milli-Q Water (Milli-Q Plus) was used for synthesis and characterization.

### 2.2. Methods

#### 2.2.1. cHANPs Production

The production of crosslinked Hyaluronic Acid Nanoparticles is a consolidate process [27–29]. Briefly, nanoprecipitation is implemented in an X-junction chip by a hydrodynamic flow focusing regime. The solvent solution of HA (50 kDa, 0.05% w/V), Gd-DTPA (0.1% w/V), and NaOH (0.025 M) is fed into the middle channel at 27 *μ*L/min, and the nonsolvent solution made of acetone and DVS (4M) is fed into the side channels at 110 *μ*L/min. The sample is collected in 20 mL of acetone and left to stir on the wheel overnight.

Purification is performed by solvent gradient dialysis, dialyzing first against increasing gradients of acetone/ethanol and then gradually against water, dropping the suspension in Spectra/Por Cellulose Membrane (MWCO 25–50 kDa), as previously reported [29].

#### 2.2.2. Anti-CD36 (Ab36) Conjugation Protocol

The antibody anti-CD36 is conjugated on the surface of the nanoparticle using two different strategies. In the first strategy, carboxyl groups of HA are activated by the addition of EDC (0.02 M) and NHS (0.01 M) and left to react for 10 min on the wheel stirrer. After streptavidin (STV) (25 *μ*g/mL) is added to NP suspension and wheel-stirred for 1 h. Excess of streptavidin is removed by centrifugation in Corning® Spin-X® UF Concentrators with a MWCO of 50 kDa by Corning (New York, USA) at 2000 rpm, 4°C for 10 min.

The BCA assay is used to measure the amount of STV bound to ST-cHANPs. For quantification, 150 *μ*L of NPs and 150 *μ*L of buffer are put in a 96-well plate and left to react for 1 h at 60°C. The amount STV is quantified through a calibration curve (0.5–30 *μ*g/mL). The absorbance of bare cHANPs is measured for each batch at least in triplicate, and its value is subtracted from the total absorbance.

Then, the biotinylated anti-CD36 (0.005 *μ*g/mL) is added to the streptavidin-conjugated nanoparticles (ST-cHANPs) and left to react for 4 h. The unreacted antibody is removed by centrifugation in Corning Concentrators with a MWCO of 50 kDa by Corning (New York, USA) at 3000 rpm, 4°C for 10 min. In the following sections, this formulation is reported as Ab36-ST-cHANPs.

In the second strategy, the anti-CD36 is added directly to the NPs surface. Carboxyl groups of HA are activated by the addition of EDC (0.02 M) and NHS (0.01 M) and left to react for 10 min on the wheel stirrer. Afterward, anti-CD36 (0.0025 *μ*g/mL) is added directly to the suspension and left to react for 4 h. The unreacted antibody is removed by centrifugation in Corning Concentrators with a MWCO of 50 kDa by Corning (New York, USA) at 3000 rpm, 4°C for 10 min. In the following section, this formulation is reported as Ab36-cHANPs.

### 2.3. Morphological Characterization of cHANPs, Ab36-ST-cHANPs, and Ab36-cHANPs

Size and polydispersity of the bare and conjugated cHANPs are measured by dynamic light scattering (DLS) by Zetasizer Nano, Malvern Panalytical (UK). The size measurement is performed dropping 1 mL of suspension in a square glass cuvette (Optical Cuvette, Sarstedt) at room temperature in triplicate.

Scanning electron microscopy (SEM), Carl Zeiss Ultraplus Field Emission, Zeiss (Germany) and transmission electron microscopy (TEM), Cryo-TEM TECNAI G2-20, and FEI (OR, USA) observations are performed to investigate the nanoparticle morphology.

For SEM observations, 200 *μ*L of suspension is deposited by ultrafiltration on polycarbonate Isopore™ membrane filters with a 50 nm cut-off by Merck KGaA (Germany) then left to dry. A 7 nm Au layer is deposited via sputter coating on the membrane filter that is observed at 10 kV. 20 *μ*L of suspension is dropped on copper grids with carbon film by Agar Scientific Ltd. (Stansted, UK) and left to dry prior to observation at 120 kV.

#### 2.3.1. Gd-DTPA Quantification of cHANPs, Ab36-ST-cHANPs, and Ab36-cHANPs

To quantify the amount of Gd-DTPA in all the different formulations of NPs, Inductively Coupled Plasma ICP-MS NexION 350 measurements, PerkinElmer Inc. (Waltham, MA, USA), are performed. Samples are diluted to 1:250 and measured in triplicate.

All the data related to measurement are collected by the interface Syngistix Nano Application Module. In the Gd-DTPA measurement, *m*/*z* 157, 100 *μ*s dwell time, and no settling time are set.

#### 2.3.2. Relaxometric Properties of cHANPs, Ab36-ST-cHANPs, and Ab36-cHANPs

Longitudinal relaxation times are measured on all formulations, and results are compared to free Gd-DTPA solutions at similar concentrations. For the measurement, the samples are vigorously stirred, and 300 *μ*L of the suspension is dropped in glass tubes. The changes in longitudinal relaxation time (T1) at 1.5 Tesla and 37°C are measured by Minispec Benchtop Relaxometer (Bruker Corporation). The distribution of relaxation times is obtained by a CONTIN algorithm, and the integral of a peak is attributed to the contribution of the measured species to the relaxation time spectrum.

#### 2.3.3. Morphological Characterization of Atherosclerotic Plaques (AP) by Electron Microscopy (EM)

Atherosclerotic plaques (APs) are harvested by carotid endarterectomy from subjects with severe atherosclerotic disease (>70% stenosis or 50% to 70% stenosis with clinical symptoms—according to the American Heart Association guidelines) and donated for research with the written consent by 6 male patients recruited by the Department of Public Health (Vascular Surgery Unit) of the University of Naples Federico II (Ethical Committee of the University of Naples Federico II-Number 157/13, September 9, 2013). APs are collected and transported in saline solution at 4°C, within 2 hours of explantation. If not used right after the removal, APs are stored in liquid nitrogen and progressively defrosted in 24 h prior to use.

The morphology of atherosclerotic plaques (AP) is investigated by both SEM and TEM. After macroscopic analysis and classification, atherosclerotic plaques containing some adherent intima and media are dissected and subsequently treated for electron microscopy imaging. For the TEM observation, samples are cut into pieces less than 1 mm^3^ and fixed with 0.5% glutaraldehyde plus 4% paraformaldehyde in 0.1 M sodium cacodylate overnight at 4°C then washed three times with 0.1 M sodium cacodylate buffer. APs are then postfixed with 1% osmium tetroxide/1% potassium ferrocyanide mixing in 0.1 M sodium cacodylate, for 1 hour in ice, in the dark, and washed in a chilled buffer. The en-bloc staining is performed with 4% uranyl acetate aqueous solution overnight at 4°C followed by washing in chilled water. The dissected pieces are dehydrated with an ascendant series of ethanol (30%-50%-70%-95%-absolute) on ice. Dehydrated samples for TEM imaging are embedded in Spurr epoxy resin and after polymerization at 60°C for 72 h were sectioned by Ultramicrotome (FC7-UC7, Leica). The 70 nm slices obtained are seeded on 200 mesh copper grids, and imaging is carried out by using a Tecnai G2-20 (FEI, USA) at a 120 kV, in a range of magnification between 2 *μ*m and 500 nm. Dehydrated samples intended for SEM imaging are subjected to Critical Point Drying (CPD) process before the imaging. A layer of 20 nm of gold is sputtered (HR 208, Cressington) before imaging with a Field emission SEM (Ultraplus, Zeiss, Germany). The secondary electron detector is used, and the images were acquired at 10 kV in a range of magnification between 2 *μ*m and 200 *μ*m.

#### 2.3.4. Ex Vivo Magnetic Resonance Imaging (MRI)

Precontrast and postcontrast imaging are performed on APs at 3 T on a PET/MRI (Siemens) to reconstruct tissue three-dimensional structure and evaluate contrast agent performance. Prior to imaging, each AP is submerged in Fomblin® to preserve tissue hydration and structure during imaging. Fomblin does not have a residual MR signal and does not interfere with the biological tissue signal [30]. Each experiment is performed by placing six APs in a 6-well plate, one per well. APs are divided into three groups of two to account for the intrinsic variability in terms of anatomy and composition characterizing each plaque. A typical experimental setting is presented in Figure S4. Each group of two plaques is injected with a different contrast agent: Gd-DTPA, cHANPs, and Ab36-cHANPs. By means of a cannula needle (24 G × 19 mm) fixed in the lumen of the aorta, 150 *μ*L of contrast agent suspension at the same Gd-DTPA concentration is slowly injected, and imaging is performed soon after, 15 and 30 minutes after the injection. The T1-weighted MR images of HA NPs, unloaded and loaded with Gd-DTPA at different concentrations using an inversion recovery sequence, are measured with the following parameters: TR = 2500 ms; TE = 12 ms; TI = 50, 100, 200, 400, 800, 1100, and 1800 ms; FOV = 180 × 146 mm; slice thickness = 4 mm; and acquisition matrix = 360 × 292. Each acquisition has a duration of 12 minutes.

#### 2.3.5. MRI Postprocessing

Images obtained by bidimensional *T*1-weighted spin-echo sequences are postprocessed with a software tool named MRmap to build a *T*1 map of the tissue. Maps are calculated by selecting the single sequences with the different *T*1 values in the various phases before and after injection of contrast agent. *T*1 values are measured in 20-pixel-wide Region of Interest (ROI) which are chosen in a region with apparent homogeneous contrast. *T*1 values are then normalized as follows:

(1) $$\begin{array}{c} \text{normalized }T1=\frac{T1_{\text{pre}-\text{injection}}-T1_{\text{post}-\text{injection}}}{T1_{\text{pre}-\text{injection}}}, \end{array}$$

to evaluate the contribution of every injected CA to the variation in measured *T*1.

#### 2.3.6. A Preliminary Study of the Interaction of cHANPs and Ab-cHANPs with APs: Ex Vivo Electron Microscopy

After MR imaging, atherosclerotic plaques injected with different contrast agents (cHANPs and Ab36-cHANPs) are fixed and processed for electron microscopy. The site surrounding the injection by cannula needles is dissected from each plaque and processed for TEM imaging, according to the protocol described above.

## 3. Results

### 3.1. Production and Characterization of cHANPs, Ab36-ST-cHANPs, and Ab36-cHANPs

**Figure 1:**
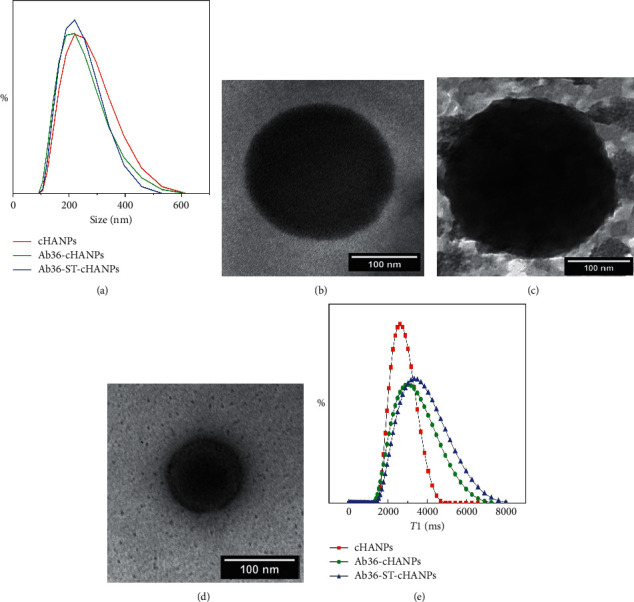
Characterization of Ab-ST-cHANPs and Ab-cHANPs. (a) Size distribution of cHANPs, Ab36-ST-cHANPs, and Ab36-cHANPs by dynamic light scattering (DLS). Transmission electron microscopy (TEM) images of (b) cHANPs, (c) Ab36-ST-cHANPs, and (d) Ab36-cHANPs. (e) Relaxometric properties of cHANPs, Ab36-ST-cHANPs, and Ab36-cHANPs by Minispec Benchtop Relaxometer.

Crosslinked Hyaluronic Acid Nanoparticles (cHANPs) are produced in microfluidics as previously reported [14, 15, 27, 28]. Briefly, an X-junction chip is used to implement a hydrodynamic flow focusing regime, and nanoparticles are produced by nanoprecipitation. The solvent phase of HA and Gd-DTPA is injected in the middle channel and the nonsolvent phase of acetone and DVS in the side channels. cHANPs have a mean size of 210 ± 65 nm as shown by particle size distribution (PSD) reported in Figure 1(a) (red line).

As discussed, HA has intrinsic AP targeting ability through CD44 receptors of endothelial cells. However, the cHANPs surface is further engineered to improve the specificity of the probe by surface decoration with the antibody anti-CD36 (Ab36) for macrophages' active targeting. The surface decoration is performed by two different strategies. In the first one, the surface of nanoparticles is conjugated to streptavidin (ST) to produce ST-cHANPs. The tetramer is covalently bound to carboxyl groups of HA, and the biotinylated form of Ab36 is conjugated exploiting the high biological affinity between ST and biotin to ST-cHANPs, producing Ab36-ST-cHANPs. The complete procedure is presented in the dedicated method section. The amount of ST successfully bound to the surface of nanoparticles is measured by BCA assay, and results show a binding efficiency of 92.6% (Table S1—Supplementary Materials). The morphology of Ab36-ST-cHANPs is investigated by TEM imaging. The comparison between Figure 1(b), which presents bare cHANPs, and Figure 1(c), which presents Ab36-ST-cHANPs, clearly shows the presence of the complex Ab36-ST on the surface of nanoparticles.

As clear from TEM images, ST, which is a big macromolecule (60 kDa), deposits in many layers on the surface of nanoparticles, probably affecting the superficial properties of cHANPs. For this reason, direct conjugation of the Ab36 to the surface of cHANPs might allow preserving the exposure of some HA chains to APs, allowing to exploit the intrinsic targeting ability of HA. In this second strategy, the amine groups of the Ab36 are covalently bound to the carboxyl group of HA to produce Ab36-cHANPs. The TEM image in Figure 1(d) reveals the presence of the Ab36 on Ab36-cHANPs. PSD of Ab36-ST-cHANPs and Ab36-cHANPs is measured by DLS and presented in Figure 1(a) (blue and green lines, respectively). The mean size of about 215.2 ± 72 nm slightly differs from cHANPs mean size. Overall, TEM images and DLS measurements demonstrate the morphological stability of nanoparticles against conjugation.

### 3.2. Relaxometric Properties of cHANPs, Ab36-ST-cHANPs, and Ab36-cHANPs: Preservation of Hydrodenticity

In the previous section, the stability of nanoparticles against conjugation in terms of size and morphology has been demonstrated. This section aims to investigate the ability of Ab36-ST-cHANPs and Ab36-cHANPs to retain Gd-DTPA after conjugation. In addition, the stability of the polymer network needs to be assessed to understand if the Hydrodenticity effect is preserved. ICP-MS measurements are performed to quantify the amount of encapsulated Gd-DTPA, and results are shown in Table S2. The measurements demonstrate that both Ab36-ST-cHANPs and Ab36-cHANPs retain Gd-DTPA with a partial loss which is more pronounced for Ab36-cHANPs (about 55%). However, the distribution of the longitudinal relaxation time *T*1 of Ab36-ST-cHANPs and Ab36-cHANPs presented in Figure 1(e) shows that, even if there is an increase in *T*1 both for Ab36-ST-cHANPs and Ab36-cHANPs with respect to cHANPs, it is more evident for Ab36-ST-cHANPs which instead retain a higher amount of Gd-DTPA. The reason for that may be found in the effect that ST has on the polymer network. The deposition of many layers of ST on the surface of nanoparticles might cause a loss of elasticity of the polymer chains as well as the hindering of efficient water exchange with Gd-DTPA, resulting in a lower relaxivity. This hypothesis can be confirmed by comparing the amount of residual Gd-DTPA in both Ab36-ST-cHANPs and Ab36-cHANPs to Gd-DTPA concentrations associated with the measured relaxivity. The comparison presented in Table S2 reveals a boosting of 4 times for cHANPs and 2.68 times for Ab36-cHANPs, confirming that this system preserves the Hydrodenticity property. Differently, no boosting is present for Ab36-ST-cHANPs, as hypothesized. For this reason, the next experiments are conducted with Ab36-cHANPs, which demonstrates the preservation of the Hydrodenticity effect.

### 3.3. Electron Microscopy Characterization of Human AP

**Figure 2:**
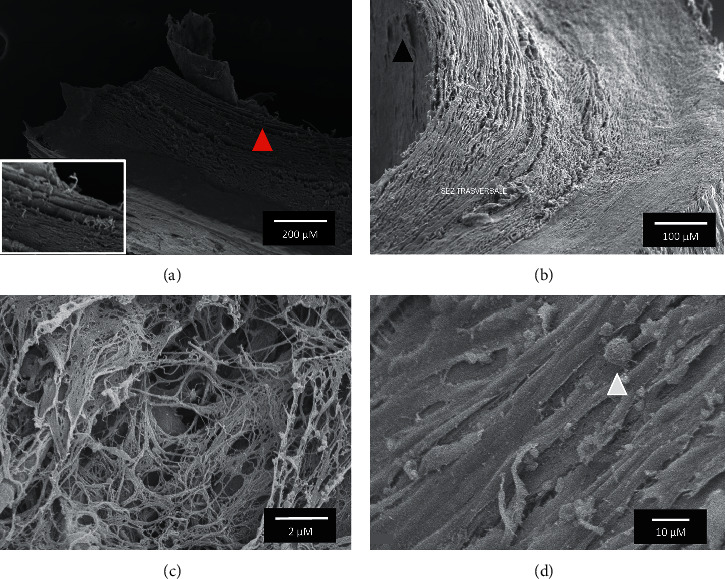
Scanning electron microscopy (SEM) of transversal sections of a human AP (a) tunica intima with detached basal lamina (red arrow head). (b) Interrupted or damaged endothelial layer (black arrow head). (c) Fibrinous reticulum in the area with absent endothelial lining. (d) Cells in the endothelial layer showing pseudopodia (white arrow head) and microvilli.

**Figure 3:**
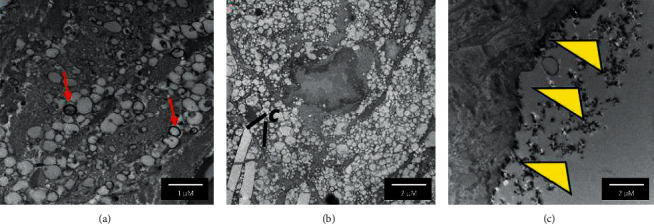
Transmission electron microscopy (TEM) of a cross section of a human AP showing (a) lipid droplets of different sizes, surrounded by calcium deposits (red arrows) and dispersed in the fibrinous reticulum. (b) Interspersed cholesterol crystals (C and black lines). (c) Damaged tunica intima (yellow arrow heads) showing the absence of endothelial cells, as observed in SEM imaging.

Atherosclerosis is activated by alterations of the endothelial layer of blood vessels [31]. For this reason, the characterization of endothelium alterations is particularly relevant to understand both the physiopathology of the disease and the composition of the tissue interacting with the imaging probe during the diagnostic investigation when injected into blood circulation. In this work, both scanning and transmission electron microscopy (SEM and TEM) are used to characterize human carotid APs and in particular their luminal wall.  Figure 2 shows SEM images of a transversal section of a human AP. Each sample analyzed has disrupted endothelial lining and presents delamination in different layers. Indeed, as shown in Figures 2(a) and 2(b), the endothelium of tunica intima is detached from the basal lamina (red arrow head), and in some areas, the endothelial lining appears to be interrupted and damaged (black arrow head). In some points, the endothelial layer is completely absent showing only the fibrinous reticulum, as presented in Figure 2(c). Also, in the areas covered by the endothelial layer, there are cells showing pseudopodia (white arrow head in Figure 2(d)) and microvilli. The formation of microvilli may indicate diffuse endo/exocytosis phenomena, confirming that dysfunctional endothelium is characterized by altered permeability.  Figure S1 shows the presence of macrophages and platelets in the tunica intima with disrupted endothelium. These observations are confirmed by TEM imaging as shown in Figure 3.  Figure 3(a) shows a cross section of human carotid AP particularly abundant in lipid droplets of different sizes and surrounded by calcium deposits (red arrow) which are mixed in the fibrinous reticulum. In Figure 3(b), the matrix reveals also the presence of interspersed cholesterol crystals which Grebe and Latz [32] defined as a hallmark of advanced atherosclerotic plaques. In Figure 3(c), the cross section of the tunica intima shows the absence of endothelial cells and thus the complete damage of the endothelial lining, as observed by SEM imaging. All these observations confirm the abundance of exposed macrophages and thus the great potential of Ab36 targeted probes for the diagnosis of APs.

### 3.4. Ex Vivo Magnetic Resonance Imaging (MRI)

**Figure 4:**
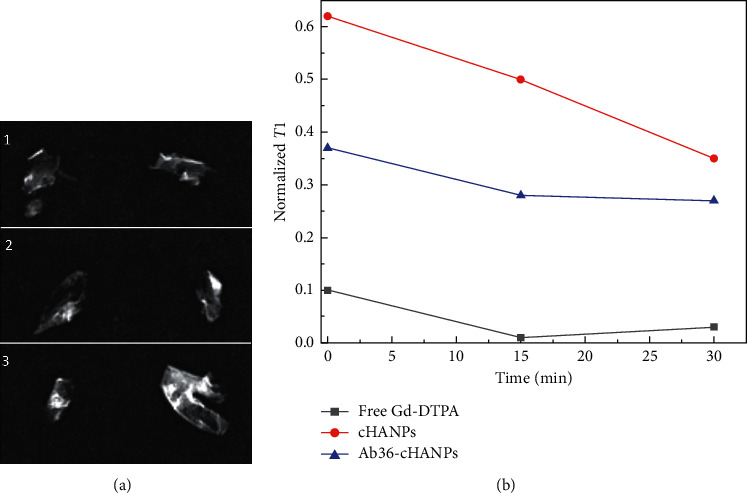
Ex vivo MRI of six APs. (a) Bidimensional MR image of APs injected with (1) free Gd-DTPA at 12 *μ*M, (2) cHANPs loaded with 12 *μ*M of Gd-DTPA, and (3) Ab36-cHANPs loaded with 10.66 *μ*M of Gd-DTPA. (b) Normalized *T*1 in the selected ROI over time.

Magnetic resonance imaging, because of its high spatial resolution, represents a promising imaging technique for the early detection of APs and determination of plaque composition. In this section, we aim to demonstrate that the Hydrodenticity of cHANPs and Ab36-cHANPs, which confers improved relaxometric properties to these imaging probes, is preserved in the complex environment of APs, demonstrating that these systems have a good potential in the enhanced MRI of atherosclerosis. In this regard, an ex vivo magnetic resonance imaging experiment is conducted on six human carotid APs injected with three different contrast agents: free Gd-DTPA, cHANPs, and Ab36-cHANPs all at a concentration of 12 *μ*m of Gd-DTPA. As known, APs have very different compositions, and to account for this variability, each probe is injected into two different plaques. The first set of experiments is dedicated to the optimization of the volume of injection to guarantee a good permeation of the tissue without liquid loss. The optimal volume of injection is set at 150 *μ*L. The anatomy of APs is reconstructed by three-dimensional inversion recovery (IR) sequences before injection of contrast agents. Afterward, *T*1-weighted images pre, post, 15 min, and 30 min after injection bidimensional spin-echo *T*1-weighted sequences are used to acquire images to build a *T*1 map of the APs, in this way evaluating the contribution of each contrast agent to AP relaxivity.  Figure 4(a) presents a typical output of the spin-echo sequences. These images are then postprocessed to build a *T*1 map as shown in Figure S2 where the value of *T*1 is evaluated in a Region of Interest (ROI) of about 20 pixels. As described in the dedicated Methods section, the contribution of each CA to the measured *T*1 is evaluated normalizing the *T*1 value measured at each time point after injection with respect to the *T*1 measured in the same ROI before injection. Results in Figure 4(b) show that both cHANPs and Ab36-cHANPs provide a normalized *T*1 significantly higher than free Gd-DTPA even if injected at the same concentration, at any time point. These results show that the Hydrodenticity of both cHANPs and Ab36-cHANPs is preserved over time in a complex environment (such as APs) despite the great variability in the composition of the injected tissues. Tables S3 and S4 in Supplementary Materials report the complete set of analyzed data.

### 3.5. A Preliminary Study of the Interaction of cHANPs and Ab36-cHANPs with APs: Ex Vivo Electron Microscopy

**Figure 5:**
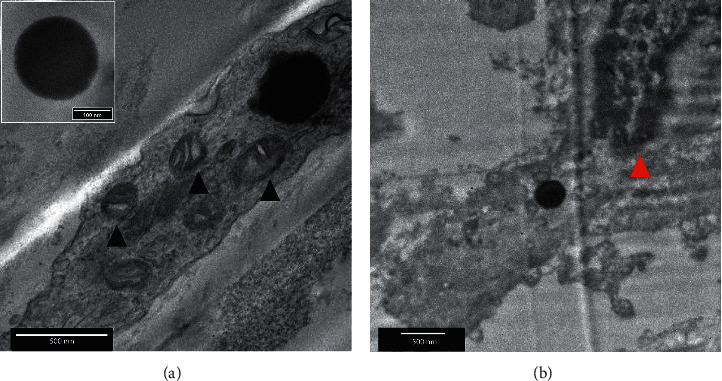
Transmission electron microscopy (TEM) of a section of a human AP injected with cHANPs and Ab36-cHANPs. Cellular localization of (a) cHANPs (black arrow head—mitochondria) and (b) Ab36-cHANPs (red arrow head—cell nucleus).

A preliminary study about the interaction of cHANPs and Ab36-cHANPs with APs is conducted by dissecting the injection site of the plaque after MRI and performing TEM imaging. In the first step, the stability of NPs at exposure to chemical reagents used for EM preparation protocol is assessed and reported in Figure S3, showing that no morphological changes occur when nanoparticles are exposed to 4% paraformaldehyde, 2.5% glutaraldehyde, 2% osmium tetroxide, and 0.1 M sodium cacodylate. Then, the injection site is dissected and prepared for TEM imaging. As described in the method section, the preparation protocol for EM includes many washing steps which we hypothesize cause the removal of the majority of the particles that are not specifically interacting with the AP. Figures 5(a) and 5(b) present TEM images of a section of AP previously injected with cHANPs and Ab36-cHANPs, respectively. A comparison with other works based on the study by Grimaldi et al. [21] on macrophage morphology confirms, in both cases, that the nanoparticles lie in cellular compartments, which can be easily recognized by the presence of mitochondria in the first image and of the cell nucleus with DNA in the second one. No particles are found in the extracellular matrix. We can hypothesize that both these observations might be due to an interaction between hyaluronic acid and scavenger receptors for cHANPs and additionally to the antigen CD-36 in the case of Ab36-cHANPs. However, preliminary results should be confirmed by a chemical assay (e.g., immunohistochemistry).

## 4. Conclusions

This work demonstrates that Hydrodenticity exploited through the crosslinked Hyaluronic Acid Nanoparticles (cHANPs) is preserved also in very complex physiological environments, such as the atherosclerotic plaques, and describes which role it can play in the precision imaging of cardiovascular diseases. In detail, the ability of the cHANPs to boost the MRI signal when injected into the AP was investigated, and localization in selected compartments was observed. Particularly, results showed that despite the great anatomical variability of the injected tissues, both cHANPs and Ab36-cHANPs produced a decrease in *T*1 more than free Gd-DTPA at the same concentration. This result confirms that thanks to the Hydrodenticity of the probes, cHANPs and Ab36-cHANPs can provide better performance than free administered Gd-DTPA, providing imaging with higher contrast. In addition, the surface functionalization with the antibody Anti-CD36 contributed to improving the specificity of the probe, accumulating selectively in cellular compartments of the tissue and potentially improving the quality of acquired images while reducing systemic toxicity.

## Data Availability

Images and tables used to support the findings of this study are available in the Supplementary Materials file.

## Supplementary Materials

Supplementary Materials
*Supplementary 1*. Table S1—BE% of ST on cHANPs. Table S2—relaxation properties of cHANPs, Ab-ST-cHANPs, and Ab-cHANPs. Table S3—pixel intensities in selected ROIs of two different experiments. Table S4—normalized *T*1 values in the ROI. *Supplementary 2*. Figure S1—SEM images of the tunica intima. Disrupted endothelial lining reveals the presence of macrophages, leukocytes, and platelets. Figure S2—*T*1 maps of APs before and after injection of (1) free Gd-DTPA, (2) cHANPs, and (3) Ab-cHANPs. (a) Preinjection. (b) Right after injection. (c) 15 min after injection. (d) 30 min after injection. Figure S3—cHANPs stability at treatment with chemical reagents used for preparation of biological samples for EM. (a) Control. (b) 4% paraformaldehyde. (c) 2.5% glutaraldehyde. (d) 2% osmium tetroxide. (e) 0.1 M sodium cacodylate. Figure S4—preparation of a typical experiment of ex vivo MRI.
