# Supplementary material for: Targeting Nanostrategies for Imaging of Atherosclerosis
Source: Contrast Media Mol Imaging. 2021 Mar 31;2021:6664471. doi: 10.1155/2021/6664471 (PMC8032543; doi:10.1155/2021/6664471)
Supplement: Supplementary Materials — Supplementary 1. Table S1—BE% of ST on cHANPs. Table S2—relaxation properties of cHANPs, Ab-ST-cHANPs, and Ab-cHANPs. Table S3—pixel intensities in selected ROIs of two different experiments. Table S4—normalized T1 values in the ROI. Supplementary 2. Figure S1—SEM images of the tunica intima. Disrupted endothelial lining reveals the presence of macrophages, leukocytes, and platelets. Figure S2—T1 maps of APs before and after injection of (1) free Gd-DTPA, (2) cHANPs, and (3) Ab-cHANPs. (a) Preinjection. (b) Right after injection. (c) 15 min after injection. (d) 30 min after injection. Figure S3—cHANPs stability at treatment with chemical reagents used for preparation of biological samples for EM. (a) Control. (b) 4% paraformaldehyde. (c) 2.5% glutaraldehyde. (d) 2% osmium tetroxide. (e) 0.1 M sodium cacodylate. Figure S4—preparation of a typical experiment of ex vivo MRI. [file 6664471.f1.docx]

# Supporting Data

## ST Conjugation

The conjugation of streptavidin (ST) to cHANPs is optimized by testing the addition of different ST concentrations to NPs. Results reported in Table S1 shows that the addition of 30 ug/mL of ST guarantees the best binding efficiency (BE%) where

$$BE\%= \frac{Measured ST}{Theoretical ST}\cdot100$$

In addition, Increasing the concentration of streptavidin does not result in a much higher concentration measured in the purified sample.

Table S1 – BE% of ST on cHANPs

|  | Streptavidin Theoretical Concentration [ug/mL] | Measured Streptavidin Concentration [ug/mL] | BE% |
| --- | --- | --- | --- |
| ST-cHANPs_1 | 30 | 27,8 | 92,6 |
| ST-cHANPs_2 | 50 | 35 | 70 |
| ST-cHANPs_3 | 100 | 37 | 37 |

## AP characterization at EM


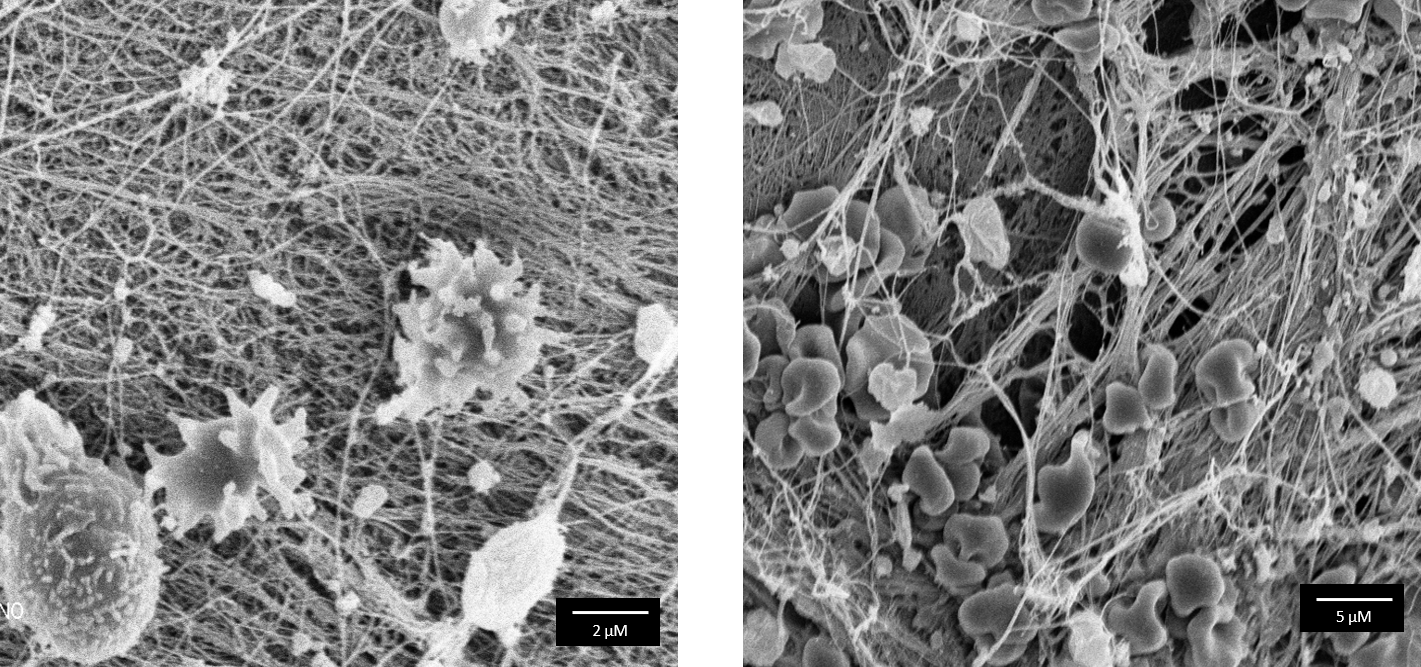


Figure S1 – SEM images of the tunica intima. Disrupted endothelial lining reveals the presence of macrophages, leukocytes and platelets.

## Relaxometric properties of cHANPs ST-cHANPs and Ab-cHANPs: preservation of Hydrodenticity

The measured amount of Gd-DTPA in nanoparticles is compared with the amount of Gd-DTPA corresponding to the measured T1 when solutions of free Gd-DTPA are measured. Table S2 presents the corresponding results.

Table S2 – Relaxation properties of cHANPs, Ab-ST-cHANPs and Ab-cHANPs

|  | **Gd-DTPA content [uM]** | **Gd-DTPA [uM]**  **T1 associated** | **Boosting Times** |
| --- | --- | --- | --- |
| cHANPs | 19 | 74,5 | 4 |
| Ab-ST-cHANPs | 18,12 | 19 | 1,04 |
| Ab-cHANPs | 10,6 | 27,9 | 2,63 |

## Ex-vivo Magnetic Resonance Imaging (MRI)


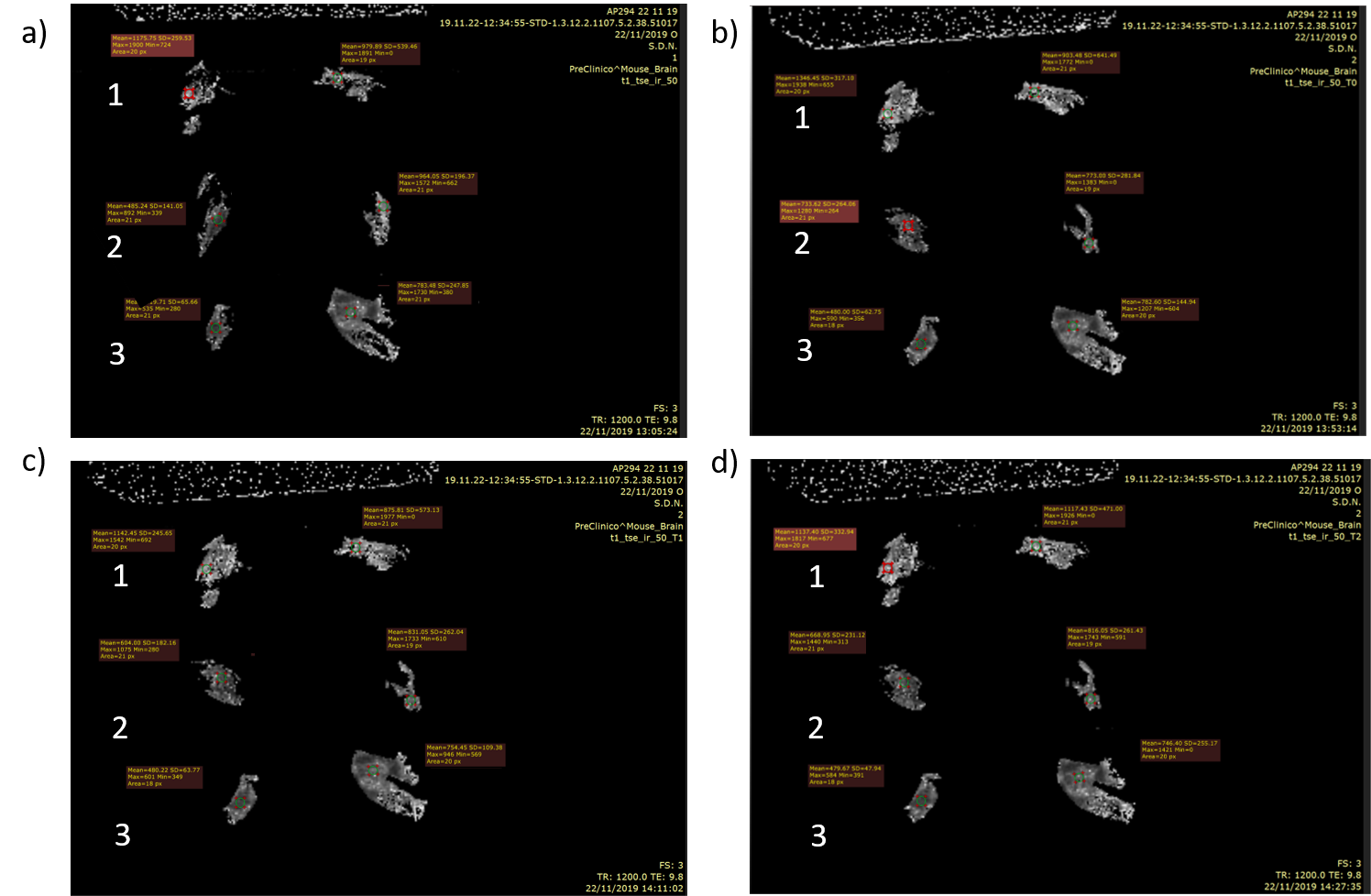


*Figure S2 – T1 maps of APs pre- and post-injection of (1) free Gd-DTPA, (2) cHANPs, (3) Ab-cHANPs. a) pre-injection; b) right after injection; c) 15 min post-injection; d) 30 min post injection.*

Table – S3 Pixel intensities in selected ROIs of two different experiments

| **^Experiment 1^** | **Gd-DTPA 12 uM** | **Gd-DTPA 12 uM_2** | **cHANPs**  **(12 uM)** | **cHANPs_2**  **(12 uM)** | **Ab-cHANPs (10,6 uM)** | **Ab-cHANPs**  **(10,6 uM)** |
| --- | --- | --- | --- | --- | --- | --- |
| Pre-injection | 1175,75 | 979,89 | 964,05 | 485,24 | 783,48 | 419,71 |
| St Dev | 259,53 | 593,86 | 196,37 | 141,05 | 247,85 | 65,66 |
| Post-injection | 1346,45 | 903,48 | 773 | 733,62 | 782,6 | 480 |
| St Dev | 317,1 | 641,49 | 281,84 | 264,06 | 144,94 | 62,75 |
| 15 min post-injection | 1142,45 | 875,81 | 831,05 | 604 | 754,45 | 480,22 |
| St Dev | 245,65 | 573,13 | 262,04 | 182,16 | 109,38 | 63,77 |
| 30 min Post-injection | 1137,4 | 1117,43 | 816,05 | 668,95 | 746,4 | 479,67 |
| St Dev | 332,94 | 471 | 261,43 | 231,12 | 255,17 | 47,94 |

| **^Experiment 2^** | | **Gd-DTPA (12 uM)** | | **Gd-DTPA_2**  **(12 uM)** | **cHANPs (12 uM)** | **cHANPs_2**  **(12 uM)** | **Ab-cHANPs (10,6 uM)** | **Ab-cHANPs**  **(10,6 uM)** |
| --- | --- | --- | --- | --- | --- | --- | --- | --- |
| Pre-injection | 378,17 | | 893,8 | | 1431,25 | 711,55 | 1210,74 | 516,1 |
| St Dev | 50,11 | | 215,61 | | 258,2 | 457,91 | 420,38 | 93,65 |
| Post-injection | 347,68 | | 810,94 | | 1500,39 | 1156,11 | 1118,95 | 704,59 |
| St Dev | 86,4 | | 387,64 | | 470,06 | 296,72 | 371,42 | 234,77 |
| 15 min post-injection | 378,68 | | 734,5 | | 1024,17 | 1031,37 | 1221,86 | 643,91 |
| St Dev | 129,78 | | 394,05 | | 567,68 | 366,27 | 350,97 | 170,93 |
| 30 min Post-injection | 370,11 | | 872,2 | | 1196,67 | 907,26 | 1347,81 | 635,64 |
| St Dev | 130,21 | | 387,87 | | 546,8 | 478,34 | 280,94 | 167,78 |

Table S4. Normalized T1 values in the ROI of Experiment 2

|  | **Gd-DTPA (12 uM)** | **Gd-DTPA_2**  **(12 uM)** | **cHANPs (12 uM)** | **cHANPs_2**  **(12 uM)** | **Ab-cHANPs (10,6 uM)** | **Ab-cHANPs**  **(10,6 uM)** |
| --- | --- | --- | --- | --- | --- | --- |
| Pre-injection | 0 | 0 | 0 | 0 | 0 | 0 |
| Post-injection | 0,080625116 | -0,092705303 | 0,048307424 | 0,624776896 | -0,075813139 | 0,365219919 |
| 15 min post-injection | 0,0013486 | -0,178227791 | 0,284422707 | 0,449469468 | 0,009184466 | 0,247645805 |
| 30 min post-injection | 0,021313166 | -0,02416648 | -0,16389869 | 0,275047432 | 0,113211755 | 0,231621779 |

# cHANPs stability at treatment with EM reagents


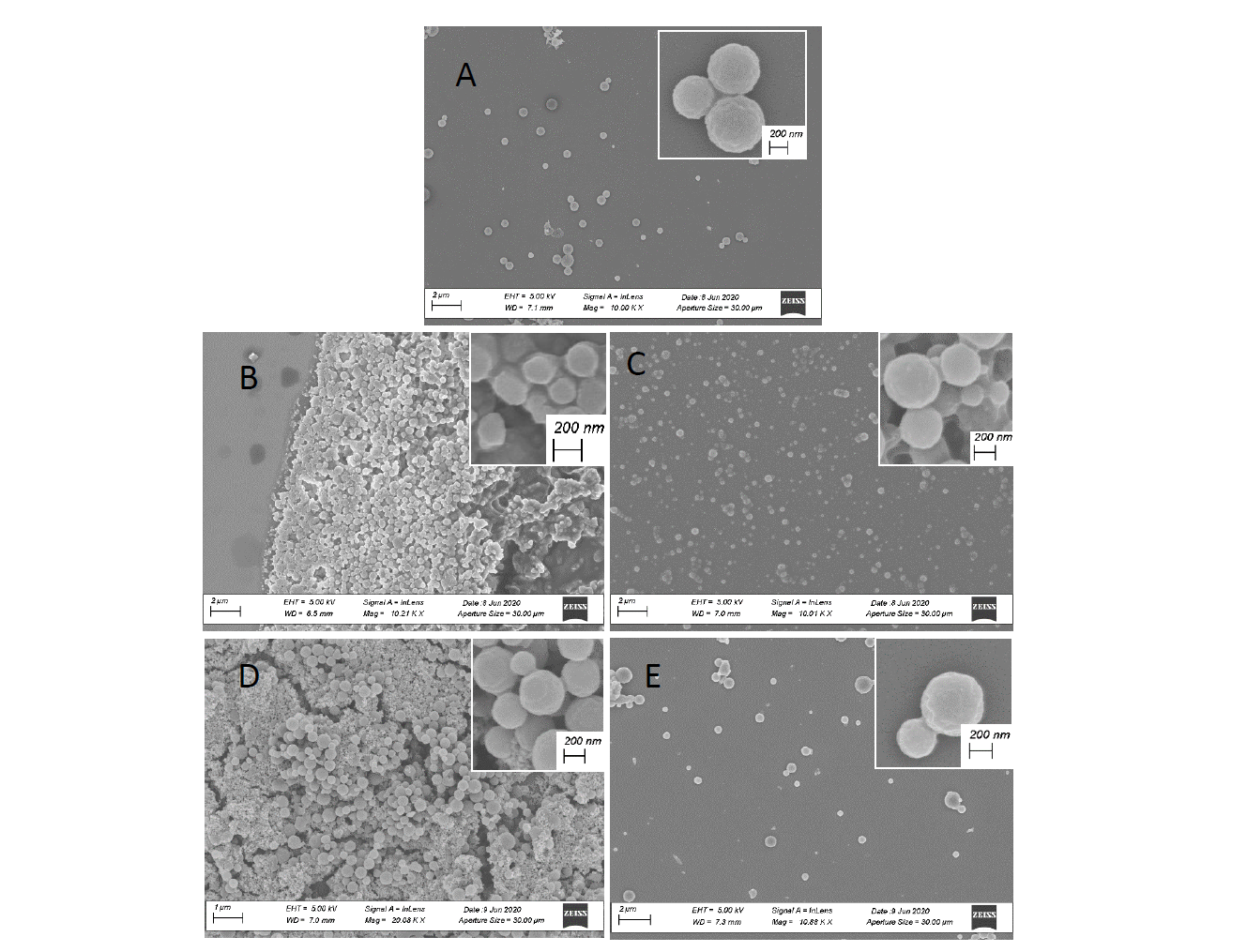


Figure S3 – cHANPs stability at treatment with chemical reagents used for preparation of biological samples for EM. A) Control B) 4%paraformaldehyde C) 2.5% glutaraldehyde D) 2% Osmium tetroxide E) 0.1M sodium cacodylate


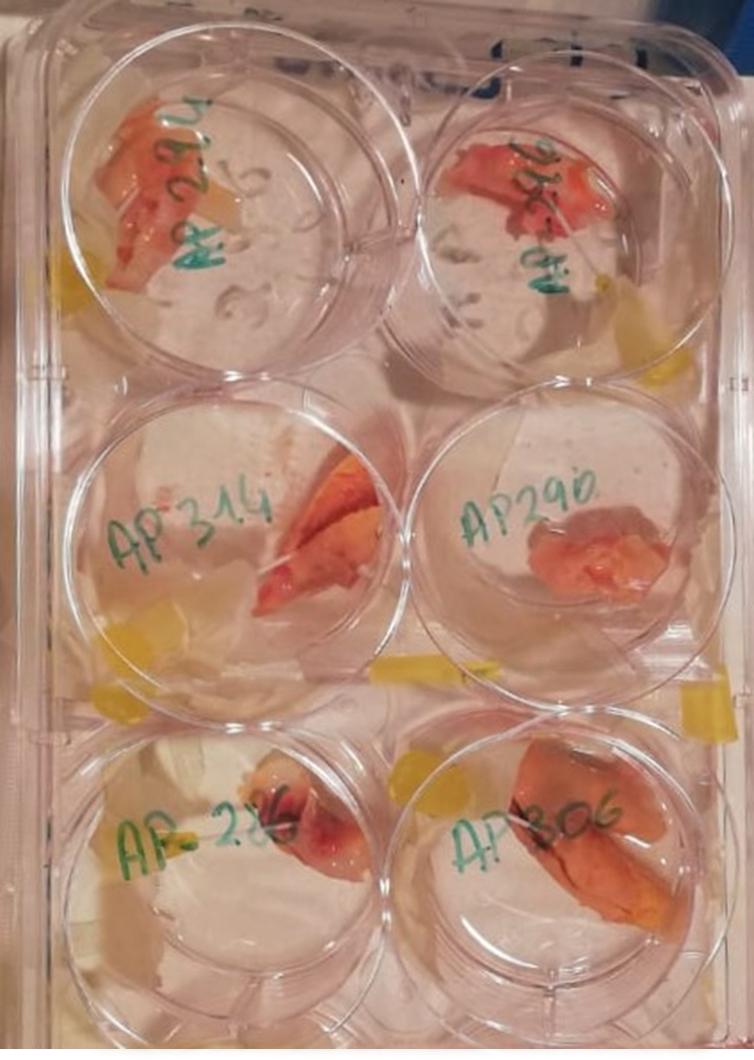


Figure S4 – Preparation of a typical experiment of ex-vivo MRI
